# BMI, Vitamin D Status, and Biochemical Markers of Mineralization in Children with Low-Energy Fractures: A Prospective Case–Control Study

**DOI:** 10.3390/children13091276

**Published:** 2026-09-20

**Authors:** Andrea Cosentino, Daniel Weghuber, Pier Francesco Indelli, Nicole Fantini, Wilhelm Berger, Olaf Stefan Schmidt, Wolfgang Högler

**Affiliations:** 1 Doctoral Program Medical Science, Paracelsus Medical University, Strubergasse 21, 5020 Salzburg, Austria; 2 Dolomiti Sportclinic, Via J.B. Purger 181, 39046 St. Ulrich, Italy; 3 Department of Orthopedics and Traumatology, Hospital of Merano (SABES-ASDAA), Teaching Hospital of the Paracelsus Medical University, 39012 Merano-Meran, Italy; wilhelm.berger@sabes.it (W.B.); olafstefan.schmidt@sabes.it (O.S.S.); 4 Department of Pediatrics, Paracelsus Medical University, Strubergasse 21, 5020 Salzburg, Austria; d.weghuber@salk.at; 5 School of Medicine, University of Trento, Via S. Maria Maddalena 1, 38122 Trento, Italy; pindelli@stanford.edu; 6 Institute of Biomechanics, Paracelsus Medical University, Strubergasse 21, 5020 Salzburg, Austria; 7 The Breyer Center for Overseas Studies in Florence, Stanford University, 50125 Florence, Italy; 8 Department of Pediatrics, Hospital of Merano (SABES-ASDAA), Teaching Hospital of the Paracelsus Medical University, 39012 Merano-Meran, Italy; nicole.fantini@sabes.it; 9 Centre for Growth and Osteology, Department of Paediatrics and Adolescent Medicine, Johannes Kepler University Linz, Krankenhausstrasse 26-30, 4020 Linz, Austria; wolfgang.hoegler@kepleruniklinikum.at

**Keywords:** pediatric fracture, adiposity, body mass index, vitamin D, bone mineralization, parathyroid hormone, alkaline phosphatase, low-energy trauma, calcium metabolism, pediatric bone health

## Abstract

**Highlights:**

**What are the main findings?**

- Higher BMI percentile and lower serum 25OHD were independently associated with fracture status after low-energy trauma.
- Vitamin D deficiency and a coherent biochemical pattern of impaired mineralization were uncommon, and calcium-intake tertiles were not independently associated with fracture status.

**What are the implications of the main findings?**

- The findings favor attention to BMI-related body size, without establishing a causal biomechanical mechanism when assessing pediatric low-energy fracture risk.
- Metabolic bone testing should be clinically targeted rather than routine, while remaining indicated for recurrent, vertebral, atypical, or very-low-trauma fractures or signs of systemic disease.

**Abstract:**

**Background:** Pediatric fractures are common, but the relative contributions of BMI-related body size and vitamin D–calcium metabolism remain uncertain. This study examined anthropometric and biochemical factors associated with fracture status in otherwise healthy children after low-energy trauma. **Methods:** This prospective case–control study included 100 children aged 3–15 years: 50 with radiographically confirmed fractures and 50 trauma controls without fracture. BMI and age- and sex-standardized BMI measures were recorded. Serum 25OHD, PTH, ALP, calcium, and phosphate were measured within seven days of injury, and dietary calcium intake was estimated. Associations were evaluated using correlation analyses, logistic regression, and BMI-adjusted analysis of covariance. **Results:** Fracture cases had higher BMI z-scores than controls (median 0.53 [IQR −0.53 to 1.44] versus −0.16 [IQR −0.89 to 0.38]; *p* = 0.006) and lower 25OHD concentrations. After adjustment for BMI z-score, the estimated geometric mean 25OHD concentration was 58.0 nmol/L (95% CI 53.0–64.1) in cases and 66.7 nmol/L (95% CI 60.3–73.7) in controls (*p* = 0.046). The Group × BMI z-score interaction was not significant (*p* = 0.327). Vitamin D deficiency (<30 nmol/L) affected only 3% of participants, and consistent biochemical abnormalities of mineralization were uncommon. **Conclusions:** Fracture status was associated with higher standardized BMI measures and lower 25OHD concentrations, but there was no evidence that the BMI–25OHD relationship differed by fracture status. These observational findings do not establish a predominant causal mechanism and support cautious, clinically targeted metabolic evaluation.

## 1. Introduction

Pediatric fractures represent a major public health concern, with around one-third of boys and girls sustaining at least one fracture before 17 years of age [1]. Bone strength in childhood results from the interaction between bone mineral content, microarchitecture, bone cell function, growth, muscle forces, and mechanical loading. Longitudinal data from the ALSPAC cohort demonstrated that children with lower bone mass have higher fracture risk, highlighting that skeletal fragility in youth is measurable and clinically relevant [2]. Although the growing skeleton is relatively elastic, indirect or rotational forces during apparently minor falls may still produce fractures. In the present study, low-energy trauma was operationally defined as a fall from standing height, a fall from a height not exceeding the child’s own height, or a comparable low-impact event. A single fracture following such trauma does not by itself establish skeletal fragility; rather, anthropometric, nutritional, biochemical, clinical, and fracture-history findings should be considered together.

Beyond intrinsic skeletal determinants, the rising prevalence of childhood overweight and obesity has made BMI-related fracture risk clinically relevant. Childhood obesity has more than tripled globally since the 1970s and is associated with substantial long-term metabolic, cardiovascular, and psychosocial morbidity [3,4,5].

Several large population-based analyses have demonstrated that children with obesity experience higher fracture risk and greater fracture severity, especially involving the lower extremities [6]. Increased body mass amplifies mechanical loading during falls and alters fracture patterns [6,7]. Although obese children may exhibit greater absolute bone mass and larger bone dimensions [8], skeletal strength may remain insufficient relative to body size and impact forces, thereby increasing fracture susceptibility [9,10].

Vitamin D deficiency may occur across the full range of BMI and has been investigated as a potential contributor to pediatric fracture risk [11,12]. Severe or prolonged deficiency, particularly when accompanied by inadequate calcium intake, can impair skeletal mineralization and cause rickets or osteomalacia, whereas the association between milder reductions in 25OHD and fractures in otherwise healthy children remains inconsistent [12,13,14,15,16]. Interpretation of 25OHD should therefore consider the clinical context and related measures such as PTH, ALP, calcium, phosphate, and calcium intake [13,14,15,16,17,18].

At the same time, lower circulating 25OHD concentrations are frequently observed in children with overweight or obesity because of volumetric dilution, sequestration in adipose tissue, and behavioral factors [11,12,19]. Consequently, an observed association between 25OHD and fracture status may be confounded by BMI, seasonality, and outdoor activity [6,13]. It is therefore important to distinguish absolute BMI from age- and sex-standardized BMI measures and to evaluate 25OHD after adjustment for BMI.

Current global consensus recommendations indicate that serum 25OHD concentrations above 50 nmol/L (20 ng/mL) are sufficient to prevent nutritional rickets [16].

Against this background, we compared BMI, BMI percentile, BMI z-score, serum 25OHD, PTH, ALP, calcium, phosphate, and estimated calcium intake between children with and without radiographically confirmed fractures after low-energy trauma. We also examined whether the between-group difference in 25OHD persisted after adjustment for BMI z-score and whether the relationship between BMI z-score and 25OHD differed according to fracture status.

## 2. Materials and Methods

This analysis derives from the BOOST (Bone Outcomes, Obesity, Sunlight, and Trauma in Children; ClinicalTrials.gov identifier NCT07143552) study, a prospective case–control project conducted at the Orthopedic Departments of Franz Tappeiner Hospital in Merano and Kaiser-Joseph Hospital in Brixen (South Tyrol, Italy). Between July 2024 and November 2025, children aged 3–15 years presenting to the emergency department after low-energy trauma, defined as a fall from standing height or a comparable low-impact mechanism, were consecutively screened for eligibility. Children with a radiographically confirmed fracture were assigned to the fracture group, whereas children who experienced a similar trauma mechanism without sustaining a fracture served as controls. Exclusion criteria were heritable bone fragility or rickets, chronic renal or hepatic disease, endocrine disorders known to affect bone metabolism, neuromuscular conditions, ongoing bisphosphonate or systemic corticosteroid therapy, unclear diagnoses, or incomplete blood sampling. The Ethics Committee for Clinical Trials of the Autonomous Province of Bolzano-Bozen approved the study (Opinion No. 25-2024). Written informed consent was obtained from parents or legal guardians, with assent from children where appropriate.

Obesity status was neither an inclusion nor an exclusion criterion, and children across the full range of BMI values were eligible. The target sample of 50 fracture cases and 50 controls was pragmatically determined according to the expected number of eligible presentations to the orthopedic emergency department during the recruitment period and the departmental funding available for biochemical testing. No a priori statistical sample-size calculation was performed. Eligible participants were enrolled consecutively according to their presentation to the emergency department until the target number was reached in each group. Controls were not individually matched to fracture cases by age, sex, or other characteristics.

A comparable trauma mechanism referred to the same low-energy category rather than individual matching between cases and controls. Low-energy trauma was operationally defined as a fall from standing height, a fall from a height not exceeding the child’s own height, or a comparable low-impact event. Controls were children in whom no fracture was identified during routine emergency-department assessment. The precise diagnoses of control injuries were not systematically categorized, although these presentations predominantly involved contusions or sprain-type soft-tissue injuries.

All participants underwent assessment within seven days of the traumatic event. Anthropometric measurements included height, weight, and body mass index (BMI). BMI, BMI percentile, and BMI z-score were used as indirect anthropometric indicators of adiposity; direct measures of body composition were not available. Age- and sex-specific BMI z scores and percentiles were calculated using LMS parameters from the Centers for Disease Control and Prevention 2000 growth charts [20]. Absolute BMI was reported descriptively, whereas age- and sex-standardized BMI measures were used for inferential analyses because the cohort included children aged 3–15 years. BMI percentile was selected as the anthropometric predictor in the primary logistic regression because of its clinical interpretability and because its bounded scale limited the influence of the extreme BMI z-score values observed in this cohort. BMI z-score, derived from the same CDC reference data, was used as a continuous standardized covariate in the BMI-adjusted analysis of 25OHD. These measures were considered complementary and were not interpreted as direct measurements of body fat.

Laboratory analyses comprised serum 25OHD (nmol/L), PTH (pg/mL), total ALP (U/L), calcium (mmol/L), and phosphate (mmol/L). Blood samples were collected under non-fasting conditions within seven days after injury; the time of day of sample collection was not systematically recorded. Serum 25OHD, total ALP, calcium, and phosphate were measured in the hospital clinical laboratory using an automated Cobas platform. PTH samples were kept on ice to preserve stability and were measured in EDTA plasma using a Cobas Pro analyzer (Roche Diagnostics GmbH, Mannheim, Germany). Results were interpreted according to the hospital laboratory’s age-appropriate reference intervals.

Dietary calcium intake was estimated using a brief food-frequency questionnaire originally validated in Italian adult women and completed by a parent or legal guardian [21]. For age-stratified analyses, participants were grouped a priori into 3–6, 7–10, and 11–15 years to reflect early childhood, mid-childhood, and adolescence, respectively.

Graphical analyses were performed to explore the relationships between BMI and bone-related biochemical parameters. Scatter plots were generated for BMI and serum 25OHD, serum 25OHD and PTH, and BMI and PTH. Serum 25OHD concentrations were categorized as deficiency (<30 nmol/L), insufficiency (30 to <50 nmol/L), or sufficiency (≥50 nmol/L) [16,22].

As a sensitivity analysis, serum 25OHD was dichotomized using the clinically relevant threshold of 50 nmol/L. A logistic regression model with fracture status coded as 1 was adjusted for BMI percentile, sex, and age group. Concentrations below 30 nmol/L were not modelled as a separate category because only three participants met this definition, which would have produced unstable estimates.

As an additional post hoc sensitivity analysis, the primary logistic regression was repeated after restricting the cohort to children aged 6–15 years. Fracture status was coded as 1, and sex, BMI percentile, serum 25OHD concentration, and age in years were included as predictors. Age was entered as a continuous variable because the original 3–6-year reference category was no longer appropriate in the restricted cohort.

We also examined whether participants displayed a biochemical pattern suggestive of impaired mineralization, operationalized as concurrent elevations of PTH and ALP above age-appropriate laboratory reference intervals [13]. This exploratory biochemical definition was not considered diagnostic of osteomalacia, which requires integration with clinical and radiographic findings.

To explore whether lower calcium intake and lower serum 25OHD concentrations were associated with fracture status, both variables were categorized into tertiles based on their distributions in the study population. Associations were examined using binary logistic regression adjusted for BMI and age. Multicollinearity among predictors was assessed using variance inflation factors (VIFs).

Because ALP varies substantially with age and growth and may also change after fracture, ALP values were interpreted against age-appropriate reference intervals and together with PTH. An isolated ALP elevation was not considered evidence of impaired mineralization [23,24]. Graphical analyses were used to support interpretation of the biochemical data and identify potential outliers or clustering patterns that could influence multivariable modelling.

Season of blood sampling was derived from the date of sample collection and categorized as summer (May–October) or winter (November–April).

Statistical analyses were performed using Jamovi (version 2.7, The jamovi project, Sydney, Australia; https://www.jamovi.org) [25]. Continuous variables were assessed for normality using the Shapiro–Wilk test and compared using independent-samples *t*-tests or Mann–Whitney U tests, as appropriate; categorical variables were analysed using chi-square tests. Spearman rank correlations were used to assess monotonic associations without assuming linearity or bivariate normality and to reduce sensitivity to influential extreme observations.

No a priori sample-size calculation was performed. Retrospective sensitivity analyses were conducted using the PAMLj module power-analysis module (version 1.2.0, developed by Marcello Gallucci; https://pamlj.github.io/ accessed on 17 September 2026). For the primary logistic regression model, a total sample of 100 participants, a balanced binary outcome, five model degrees of freedom, a two-sided α level of 0.05, and 80% power yielded a minimum detectable model-level R^2^ of 0.0925. For continuous comparisons between 50 fracture cases and 50 controls, the corresponding minimum detectable standardized mean difference was Cohen’s d = 0.566.

Because BMI z-scores were not normally distributed, they were summarized as medians and interquartile ranges and compared using the Mann–Whitney U test. For the BMI-adjusted analysis of 25OHD, concentrations were natural-log transformed because the residuals of the model using untransformed values deviated from normality. Analysis of covariance was performed with fracture status as the fixed factor and BMI z-score as the covariate. Adjusted means and 95% confidence intervals were back-transformed and reported as geometric means. A Group × BMI z-score interaction term was examined to assess whether the association between BMI and 25OHD differed according to fracture status.

The primary multivariable model included age group, sex, BMI percentile, and serum 25OHD on the basis of clinical relevance and the study objectives. All logistic regression models coded fracture cases as 1 and controls as 0; consequently, odds ratios greater than 1 indicate higher odds of fracture. Results are reported as regression coefficients (β), odds ratios (ORs), standard errors, Z statistics, and 95% confidence intervals (CIs).

The reporting of this study followed the Strengthening the Reporting of Observational Studies in Epidemiology (STROBE) guidelines for case–control studies [26]; the completed checklist is provided as Supplementary File S1.

This prospective observational case–control study was registered with ClinicalTrials.gov (NCT07143552; first submitted on 20 August 2025 and first posted on 27 August 2025). No intervention or exposure was assigned by the investigators.

## 3. Results

### 3.1. Cohort Characteristics and Fracture Distribution

**Figure 1:**
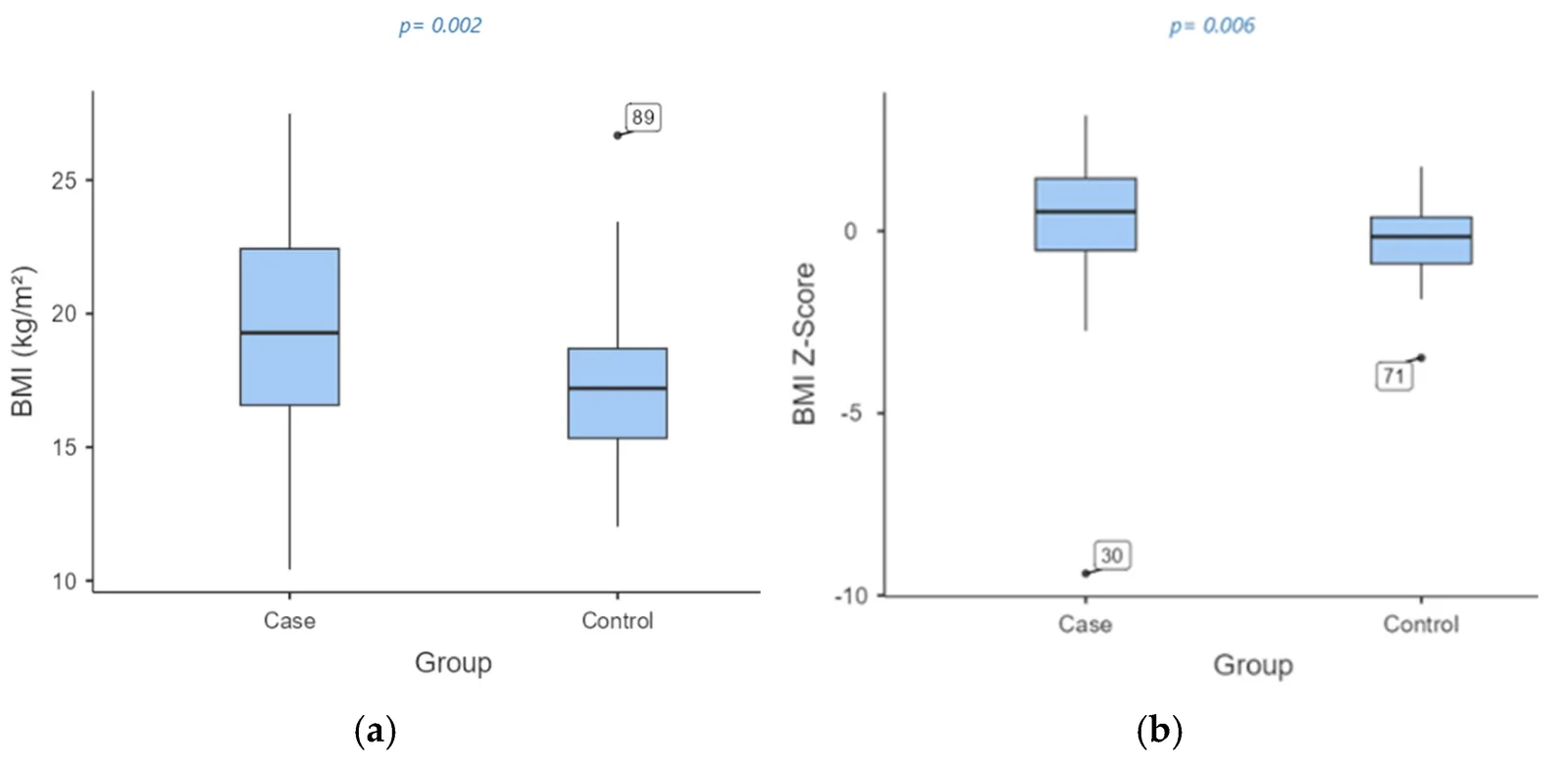
Descriptive plots of BMI (kg/m^2^) (**a**) and BMI Z-Scores (**b**) in cases vs. controls.

Of 153 children approached, five declined blood sampling, seven declined participation, 39 potential controls provided consent but did not complete blood sampling, and two children were excluded because of osteogenesis imperfecta and diabetes, respectively. The final analytical cohort therefore comprised 100 participants: 50 fracture cases and 50 controls. Fractures involved the upper extremity in 37 children and the lower extremity in 13 children. Baseline demographic, anthropometric, and biochemical characteristics are summarized in Table 1. Children with fractures had higher BMI z-scores than controls (median 0.53 [IQR −0.53 to 1.44] versus −0.16 [IQR −0.89 to 0.38]; Mann–Whitney U = 849, *p* = 0.006; Figure 1). After adjustment for BMI z-score, the estimated geometric mean 25OHD concentration was 58.0 nmol/L (95% CI 53.0–64.1) in fracture cases and 66.7 nmol/L (95% CI 60.3–73.7) in controls (F(1, 97) = 4.07, *p* = 0.046). BMI z-score was also associated with log-transformed 25OHD concentrations (F(1, 97) = 4.88, *p* = 0.029). The Group × BMI z-score interaction was not significant (F(1, 96) = 0.97, *p* = 0.327), providing no evidence that the association differed by fracture status.

Age was comparable between groups, whereas sex distribution differed significantly (Table 1).

### 3.2. Biochemical Profile and Mineralization Status

**Figure 2:**
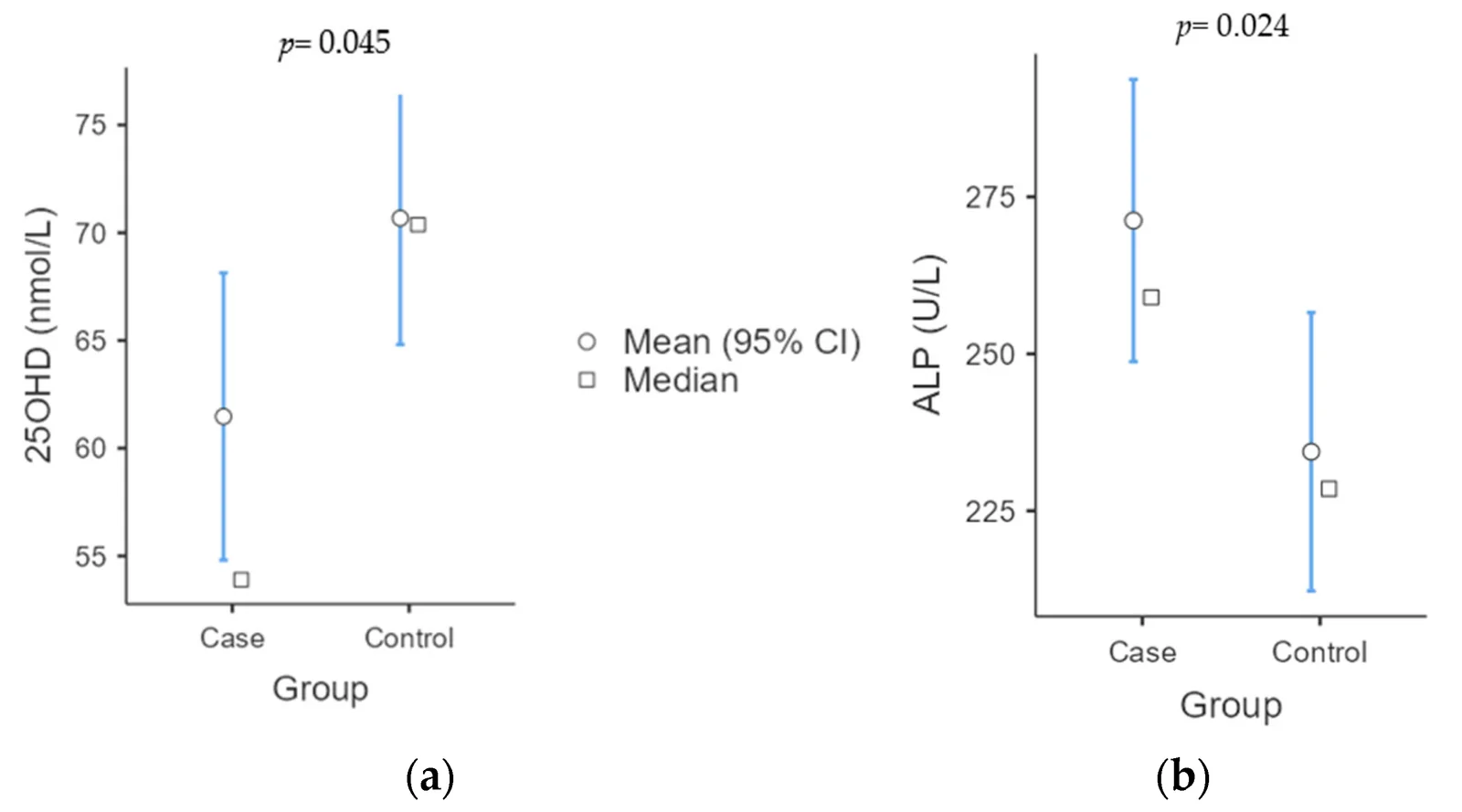
Descriptive plots of serum 25OHD (nmol/L) (**a**) and ALP (U/L) (**b**) in cases vs. controls.

**Figure 3:**
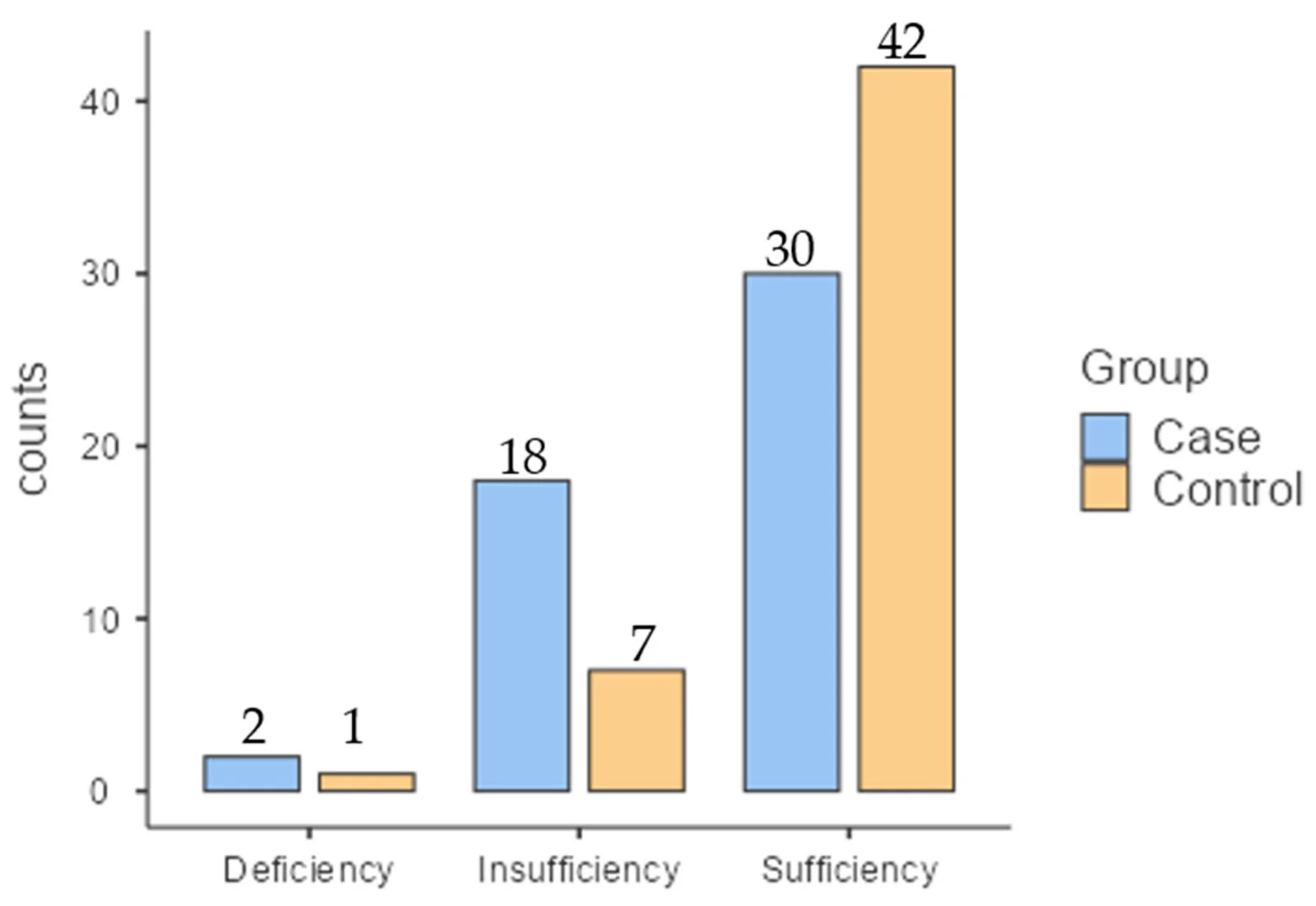
Distribution of vitamin D status categories among fracture cases and controls.

Serum 25OHD concentrations were lower in fracture cases than in controls (61.472 vs. 70.667 nmol/L; *p* = 0.045; Table 1 and Figure 2). Vitamin D deficiency (<30 nmol/L) was uncommon, occurring in three children (3% of the cohort), whereas vitamin D insufficiency (30–50 nmol/L) was more frequent. Figure 3 shows the distribution of 25OHD categories (<30, 30–50, and ≥50 nmol/L) by group.

Serum calcium, phosphate, and PTH concentrations were within age-appropriate reference ranges for most participants and did not differ significantly between groups. ALP values were higher in fracture cases than in controls (Table 1), but this finding was interpreted cautiously because samples were collected within seven days of injury and ALP may increase during early fracture healing.

Three participants (3%) had concurrent PTH and ALP values above the applicable reference intervals, constituting an exploratory biochemical pattern suggestive of impaired mineralization. This finding was not diagnostic of rickets or osteomalacia.

The distribution of blood sampling across seasons did not differ significantly between fracture cases and controls (χ^2^ = 2.58, *p* = 0.108). Among fracture cases, 54% of samples were collected during summer months (May–October), compared with 38% among controls.

Spearman correlation analyses between BMI and biochemical markers of bone mineralization are presented in Table 2. BMI was inversely correlated with serum 25OHD (ρ = −0.254, *p* = 0.011) and positively correlated with PTH (ρ = 0.268, *p* = 0.007). Serum 25OHD was inversely correlated with PTH (ρ = −0.335, *p* < 0.001). ALP showed a moderate positive correlation with PTH (ρ = 0.363, *p* < 0.001), whereas no significant correlation was observed between ALP and either 25OHD or BMI. All VIF values were <2, indicating that multicollinearity was unlikely to materially affect the regression estimates. Correlation analyses were restricted a priori to BMI and variables directly related to bone mineralization and its regulation (25OHD, PTH, and ALP), because the aim was to examine metabolic relationships rather than general demographic associations.

The vast majority of children, including those with lower serum 25OHD concentrations, did not display biochemical features suggestive of impaired mineralization.

### 3.3. Analysis of BMI and Bone Metabolism

**Figure 4:**
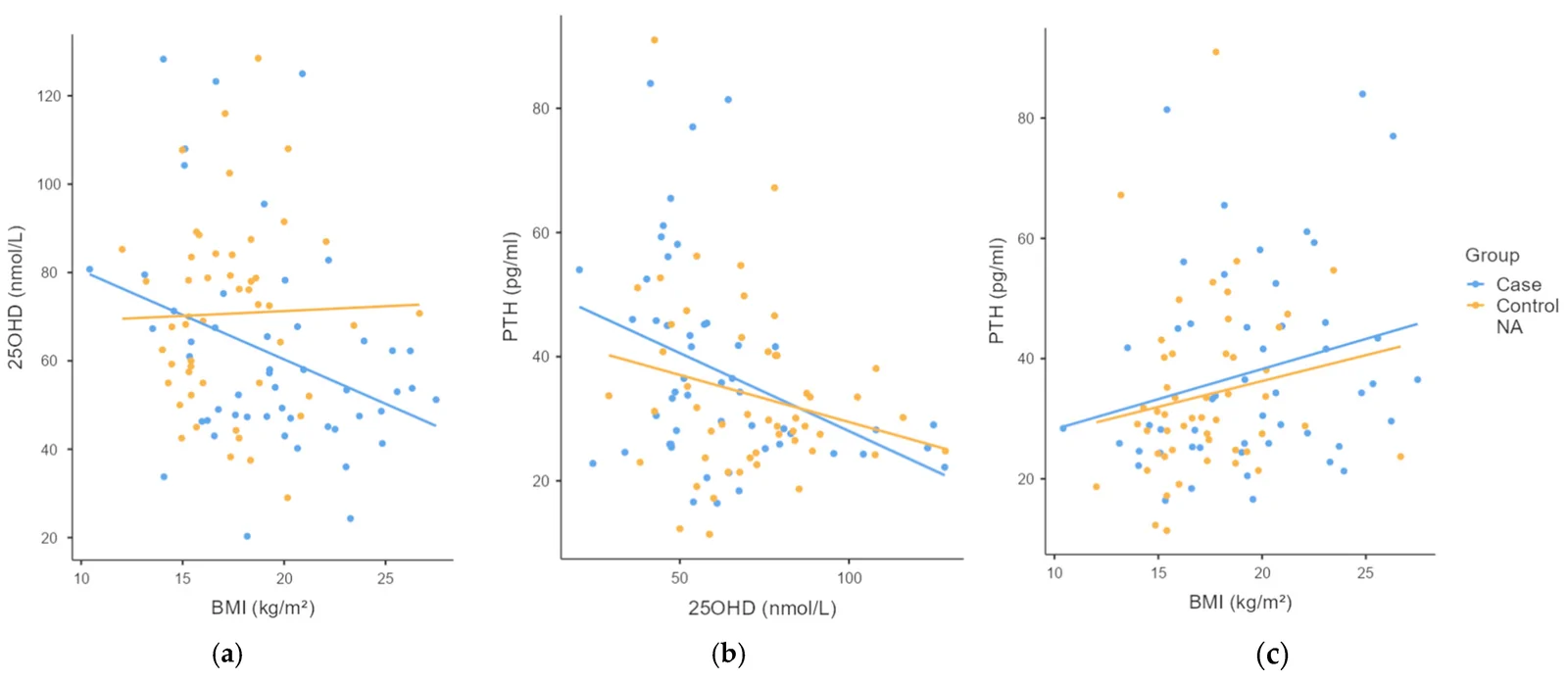
Relationships between (**a**) BMI and 25OHD, (**b**) 25OHD and PTH, and (**c**) BMI and PTH.

Scatter plots were used to visualize the relationships between BMI and serum 25OHD, serum 25OHD and PTH, and BMI and PTH (Figure 4a–c).

Visual inspection suggested an inverse relationship between BMI and 25OHD among fracture cases and a comparatively flatter trend among controls (Figure 4a). However, the formal Group × BMI z-score interaction was not significant (F(1, 96) = 0.97, *p* = 0.327). Accordingly, the apparent difference between the group-specific trends should be considered descriptive; the analysis provided no evidence that the relationship between BMI and 25OHD differed by fracture status.

An inverse relationship between 25OHD and PTH was observed in both groups, with higher PTH levels corresponding to lower 25OHD concentrations (Figure 4b). In contrast, BMI showed a slight positive association with PTH, particularly among cases (Figure 4c).

### 3.4. Multivariable Analysis of Fracture Status

The primary multivariable logistic regression model was significant (χ^2^(5) = 22.1, *p* < 0.001; Nagelkerke’s R^2^ = 0.264). Higher BMI percentile was associated with higher odds of fracture (adjusted OR 1.018 per percentile, 95% CI 1.004–1.033; *p* = 0.013), whereas higher serum 25OHD was associated with lower fracture odds (adjusted OR 0.979 per nmol/L, 95% CI 0.960–0.999; *p* = 0.040). Female participants had lower fracture odds than male participants (adjusted OR 0.269, 95% CI 0.107–0.680; *p* = 0.006). Age group did not reach overall significance in the likelihood-ratio test (*p* = 0.061), although the 7–10-year group had higher fracture odds than the 3–6-year reference group (adjusted OR 4.885, 95% CI 1.164–20.497; *p* = 0.030) (Table 3).

In the sensitivity analysis using the clinical 25OHD threshold, concentrations <50 nmol/L were associated with higher adjusted odds of fracture compared with concentrations ≥50 nmol/L (adjusted OR 3.58, 95% CI 1.26–10.11; *p* = 0.016). The overall model was significant (χ^2^(5) = 23.9, *p* < 0.001; Nagelkerke’s R^2^ = 0.283). The wide confidence interval indicates limited precision (Table 4).

The sensitivity analysis restricted to children aged 6–15 years included 85 participants. The overall model was significant (χ^2^(4) = 23.3, *p* < 0.001; Nagelkerke’s R^2^ = 0.321). Higher BMI percentile remained associated with higher odds of fracture (adjusted OR 1.017 per percentile, 95% CI 1.001–1.033; *p* = 0.044), whereas higher serum 25OHD remained associated with lower fracture odds (adjusted OR 0.970 per nmol/L, 95% CI 0.947–0.993; *p* = 0.012). Female sex was associated with lower fracture odds (adjusted OR 0.194, 95% CI 0.070–0.537; *p* = 0.002), while age was not independently associated with fracture status (*p* = 0.170).

The original exploratory analysis using cohort-derived tertiles of 25OHD and calcium intake is presented in Supplementary Table S1. Because the lowest-tertile estimate was imprecise and based on data-derived boundaries, the findings should be interpreted as exploratory.

## 4. Discussion

In this prospective case–control study, children with radiographically confirmed fractures after low-energy trauma had higher BMI-based measures and lower serum 25OHD concentrations than trauma controls without fracture. After adjustment for age group and sex, higher BMI percentile and lower 25OHD were independently associated with fracture status. Sensitivity analyses using the clinical 25OHD threshold and restricting the cohort to children aged 6–15 years yielded broadly consistent results. However, the modest sample size and observational design preclude conclusions about whether BMI-related body size or vitamin D status has a causal or predominant role in fracture occurrence.

The findings differed according to the BMI measure used. Absolute BMI was higher in fracture cases, but because it is strongly age dependent, it is unsuitable as the principal comparison in a cohort aged 3–15 years. The parametric comparison of mean BMI z-scores was not statistically significant, whereas the rank-based comparison was significant. These results are not contradictory: the *t*-test compares group means, whereas the Mann–Whitney U test compares ranks and is less affected by non-normal distributions and extreme values. Accordingly, BMI z-score was reported as median and interquartile range and compared using the Mann–Whitney U test. BMI percentile was used in the primary logistic regression because of its clinical interpretability and bounded scale, whereas BMI z-score was used as the standardized covariate in the BMI-adjusted analysis of 25OHD. These measures are complementary. Because BMI, BMI percentile, and BMI z-score cannot distinguish fat mass from lean mass, the findings concern BMI-related body size rather than directly measured adiposity.

The association between higher BMI and fracture status is consistent with previous studies reporting increased fracture risk or severity among children with overweight or obesity [6,7,9,10,27]. Proposed explanations include greater impact forces during falls, skeletal strength that is insufficient relative to body size, and differences in balance, coordination, or motor performance [7,8,9,28]. Although children with obesity may have greater absolute bone mass and larger bone dimensions, these adaptations may be insufficient relative to body mass and the forces generated during a fall [8]. However, we did not directly assess biomechanical parameters, fall characteristics, body composition, or motor performance. Fracture susceptibility has also been reported among children with low BMI [29], suggesting that skeletal vulnerability may occur at both extremes of body size. Our findings should therefore not be interpreted as evidence of a specifically biomechanical mechanism.

Serum 25OHD was inversely associated with BMI, raising the possibility that the lower concentrations in fracture cases partly reflected BMI-related volumetric dilution or associated behavioral factors [11,12,19]. Nevertheless, the between-group difference remained borderline significant after adjustment for BMI z-score. The adjusted geometric mean 25OHD concentration was lower in fracture cases, but the Group × BMI z-score interaction was not statistically significant. The analysis therefore provided no evidence that the relationship between BMI z-score and 25OHD differed by fracture status. Concentrations below 50 nmol/L were also associated with higher adjusted fracture odds, although the wide confidence interval indicated limited precision. Thus, the lower 25OHD concentration in fracture cases was not fully explained by BMI z-score, but these findings cannot establish an independent causal effect of vitamin D on fracture risk. This interpretation is consistent with the heterogeneous findings of previous pediatric studies and meta-analyses [11,12].

Severe vitamin D deficiency was uncommon, and calcium, phosphate, and PTH concentrations did not differ significantly between groups. Three participants had concurrent elevations of PTH and ALP, but this exploratory biochemical pattern was not diagnostic of rickets or osteomalacia. No consistent group-level biochemical pattern suggestive of impaired mineralization was identified. However, normal biochemical measurements do not exclude reduced bone mass, impaired bone quality, or altered bone microarchitecture. Because DXA, pQCT, and direct body-composition measurements were unavailable, the study cannot determine whether the observed associations reflect bone mass, bone geometry, bone quality, body composition, mechanical loading, or a combination of these factors.

ALP requires cautious interpretation because its concentration varies with age and growth and may also change during fracture healing [23,24,30,31]. Because blood samples were obtained within seven days after injury, the higher ALP concentration in fracture cases may partly represent an early post-injury response rather than pre-injury skeletal metabolism. ALP was therefore excluded from the revised primary multivariable model, and an isolated elevation was not interpreted as evidence of impaired mineralization. The inverse association between 25OHD and PTH was physiologically plausible and consistent with previous pediatric studies [32,33], but it does not by itself demonstrate clinically relevant impairment of skeletal mineralization.

In the analysis restricted to children aged 6–15 years, higher BMI percentile and lower 25OHD remained associated with fracture status, suggesting that the principal findings were not driven solely by the inclusion of preschool-aged children. However, this post hoc sensitivity analysis included only 85 participants and should be considered supportive rather than confirmatory. A greater proportion of fracture cases were boys. Although sex was included in the multivariable models, this imbalance may reflect unmeasured sex-related differences in physical activity, trauma exposure, or injury patterns.

The results do not support a general recommendation against metabolic testing in children with fractures. They instead support further study of a clinically targeted approach that considers trauma mechanism, fracture history and location, growth, musculoskeletal symptoms, dietary factors, chronic disease, and clinical signs of rickets or another systemic disorder [16,18]. Excessively restricting testing could result in missed diagnoses, particularly among children with recurrent, vertebral, atypical, or very-low-trauma fractures. The balance between routine and targeted assessment may also differ in populations with a higher prevalence of vitamin D deficiency, limited sunlight exposure, nutritional vulnerability, or socioeconomic disadvantage. Because this cohort was recruited in a single European region, the findings may not apply to non-European populations or healthcare settings with different demographic and nutritional characteristics.

Strengths of this study include its prospective design, consecutive recruitment, inclusion of trauma controls exposed to a comparable low-energy mechanism, and combined assessment of standardized BMI measures, estimated calcium intake, and biochemical markers. This study has several limitations. The relatively small sample size and single-region case–control design limit the precision of subgroup and exploratory analyses and do not permit causal inference. No a priori sample-size calculation was performed, and the retrospective sensitivity calculations indicate that smaller effects may have remained undetected; nonsignificant findings should therefore not be interpreted as evidence of equivalence. Biochemical measurements were obtained within seven days after trauma; therefore, post-injury changes, particularly in ALP, may have influenced the results. The time of day of blood collection was not systematically recorded, and sampling-time variation may have influenced PTH and other bone-turnover measurements. Residual confounding by pubertal stage, physical activity, sunlight exposure, motor competence, and bone mineral density cannot be excluded, while questionnaire-based calcium-intake estimates may be subject to recall and measurement bias. The calcium-intake questionnaire was originally validated in adult women rather than children. Direct measurements of body composition, biomechanical parameters, fall characteristics, bone density, and bone geometry were not available, and control injuries were not systematically categorized beyond the absence of a fracture. These findings should therefore be interpreted cautiously and validated in larger longitudinal studies.

## 5. Conclusions

In this prospective case–control study, fracture status after low-energy trauma was associated with higher BMI-based measures and lower serum 25OHD concentrations. The adjusted difference in 25OHD was borderline, and the Group × BMI z-score interaction was not significant. Severe vitamin D deficiency and a consistent biochemical pattern suggestive of impaired mineralization were uncommon. These observational findings do not establish a causal mechanism or a general screening recommendation. In this selected cohort, they support further evaluation of clinically targeted metabolic assessment.

## Figures and Tables

**Table 1:** Demographic, anthropometric, biochemical, and dietary characteristics according to fracture status- Values are presented as number (percentage), mean ± standard deviation, or median [interquartile range], as appropriate. BMI z-scores were compared using the Mann–Whitney U test; other continuous variables were compared using independent-samples *t*-tests. All *p*-values are two-sided. BMI, body mass index; 25OHD, 25-hydroxyvitamin D; PTH, parathyroid hormone; ALP, alkaline phosphatase; IQR, interquartile range.

**A. Demographic and Anthropometric Characteristics**						
**Group**	***n***	**Sex, *n* (%)**		**Age, Years**	**BMI, kg/m^2^**	**BMI z-Score**
Fracture cases	50	M: 34 (68%); F: 16 (32%)		10.19 ± 3.14	19.43 ± 3.99	0.53 [−0.53, 1.44]
Controls	50	M: 23 (46%); F: 27 (54%)		9.94 ± 3.32	17.27 ± 2.72	−0.16 [−0.89, 0.38]
*p*-value	—	**0.044**		0.695	**0.002**	**0.006**
**B. Biochemical and dietary characteristics**						
**Group**	**25OHD, nmol/L**	**PTH, pg/mL**	**ALP, U/L**	**Calcium, mmol/L**	**Phosphate, mmol/L**	**Calcium Intake, mg/day**
Fracture cases	61.47 ± 24.04	37.69 ± 16.55	271.24 ± 81.11	2.40 ± 0.10	1.42 ± 0.19	658.03 ± 264.36
Controls	70.68 ± 21.15	33.92 ± 14.37	234.40 ± 79.97	2.42 ± 0.09	1.47 ± 0.20	715.11 ± 268.73
*p*-value	**0.045**	0.226	**0.024**	0.324	0.162	0.287

**Table 2:** Spearman correlation matrix showing associations between body mass index (BMI) and biochemical markers of bone mineralization, including serum 25-hydroxyvitamin D (25OHD), parathyroid hormone (PTH), and alkaline phosphatase (ALP). Correlation coefficients (Spearman’s rho), degrees of freedom (df), and *p*-values are reported. Significance levels are indicated as * *p* < 0.05, ** *p* < 0.01, and *** *p* < 0.001.

		25OHD (nmol/L)	BMI (kg/m^2^)	PTH (pg/mL)	ALP (U/L)
**25OHD (nmol/L)**	Spearman’s rho	—			
	df	—			
	*p*-value	—			
**BMI (kg/m^2^)**	Spearman’s rho	−0.254 *	—		
	df	98	—		
	*p*-value	**0.011**	—		
**PTH (pg/mL)**	Spearman’s rho	−0.335 ***	0.268 **	—	
	df	98	98	—	
	*p*-value	**<0.001**	**0.007**	—	
**ALP (U/L)**	Spearman’s rho	0.055	0.135	0.363 ***	—
	df	98	98	98	—
	*p*-value	0.587	0.180	**<0.001**	—

**Table 3:** Multivariable binomial logistic regression of fracture status, coded as fracture case = 1 and control = 0. Male sex and age 3–6 years were the reference categories. OR, odds ratio; CI, confidence interval; SE, standard error. Overall model: χ^2^(5) = 22.1, *p* < 0.001; McFadden’s R^2^ = 0.159; Nagelkerke’s R^2^ = 0.264.

Predictor	β	SE	Z	*p*	Adjusted OR	95% CI
Intercept	0.1363	0.99879	0.137	0.891	1.146	0.162–8.117
BMI percentile (%)	0.0182	0.00727	2.497	**0.013**	1.018	1.004–1.033
25OHD (nmol/L)	−0.0210	0.01023	−2.049	**0.040**	0.979	0.960–0.999
Female vs. male	−1.3114	0.47244	−2.776	**0.006**	0.269	0.107–0.680
Age 7–10 vs. 3–6 years	1.5861	0.73172	2.168	**0.030**	4.885	1.164–20.497
Age 11–15 vs. 3–6 years	0.6673	0.65266	1.022	0.307	1.949	0.542–7.004

**Table 4:** Sensitivity logistic regression using the clinically relevant serum 25OHD threshold. Fracture status was coded as case = 1 and control = 0. Serum 25OHD ≥ 50 nmol/L, male sex, and age 3–6 years were the reference categories. ORs are adjusted for all variables included in the model. Overall model: χ^2^(5) = 23.9, *p* < 0.001; McFadden’s R^2^ = 0.172; Nagelkerke’s R^2^ = 0.283. OR, odds ratio; CI, confidence interval; SE, standard error.

Predictor	β	SE	Z	*p*	Adjusted OR	95% CI
Intercept	−1.5892	0.73740	−2.155	0.031	0.204	0.048–0.866
25OHD < 50 vs. ≥50 nmol/L	1.2739	0.53056	2.401	**0.016**	3.575	1.264–10.113
BMI percentile (%)	0.0185	0.00732	2.529	**0.011**	1.019	1.004–1.033
Female vs. male	−1.2888	0.47825	−2.695	**0.007**	0.276	0.108–0.704
Age 7–10 vs. 3–6 years	1.4972	0.74509	2.009	**0.044**	4.469	1.038–19.250
Age 11–15 vs. 3–6 years	0.6499	0.66913	0.971	0.331	1.915	0.516–7.109

## Data Availability

The data presented in this study are available from the corresponding author on reasonable request. The data are not publicly available because of privacy and ethical restrictions relating to pediatric participant data.

## Supplementary Materials

The following supporting information can be downloaded at: https://www.mdpi.com/article/10.3390/children13091276/s1, File S1: STROBE Statement—checklist of items that should be included in reports of observational studies; Table S1: Exploratory multivariable binomial logistic regression of fracture status using cohort-derived tertiles of serum 25OHD and calcium intake.

## Abbreviations

The following abbreviations are used in this manuscript:

25OHD	25-hydroxyvitamin D
ALP	Alkaline phosphatase
ALSPAC	Avon Longitudinal Study of Parents and Children
BMI	Body mass index
BOOST	Bone Outcomes, Obesity, Sunlight, and Trauma in Children
CDC	Centers for Disease Control and Prevention
CI	Confidence interval
dF	Degrees of freedom
LMS	Lambda–mu–sigma method
OR	Odds ratio
PTH	Parathyroid hormone
SD	Standard deviation
SE	Standard error
VIF	Variance inflation factor
