# Supplementary material for: BMI, Vitamin D Status, and Biochemical Markers of Mineralization in Children with Low-Energy Fractures: A Prospective Case–Control Study
Source: Children (Basel). 2026 Sep 20;13(9):1276. doi: 10.3390/children13091276 (PMC13605413; doi:10.3390/children13091276)
Supplement: Supplementary file 1 [file children-13-01276-s001.zip › Supplementary File S1.pdf]

STROBE Statement—checklist of items that should be included in reports of observational studies

	Item No.	Recommendation	Page No.	Relevant text from manuscript
**Title and abstract**	1	(*a*) Indicate the study's design with a commonly used term in the title or the abstract	1	Title: '...a prospective case-control study'.
		(*b*) Provide in the abstract an informative and balanced summary of what was done and what was found	1-2	Structured Abstract reports the background, methods, principal results, and conclusions.
**Introduction**				
Background/rationale	2	Explain the scientific background and rationale for the investigation being reported	2-3	Introduction: fracture epidemiology, adiposity, vitamin D, and mineralization rationale.
Objectives	3	State specific objectives, including any prespecified hypotheses	3	'Against this background, we evaluated anthropometric and biochemical differences...'
**Methods**				
Study design	4	Present key elements of study design early in the paper	3	'...a prospective case-control project...'
Setting	5	Describe the setting, locations, and relevant dates, including periods of recruitment, exposure, follow-up, and data collection	3	Two South Tyrol hospitals; July 2024-November 2025; assessment within seven days.
Participants	6	(*a*) *Cohort study*—Give the eligibility criteria, and the sources and methods of selection of participants. Describe methods of follow-up<br>*Case-control study*—Give the eligibility criteria, and the sources and methods of case ascertainment and control selection. Give the rationale for the choice of cases and controls<br>*Cross-sectional study*—Give the eligibility criteria, and the sources and methods of selection of participants	3	Age 3-15 years; consecutive emergency-department screening; radiographic fracture cases; similar-trauma, non-fracture controls; exclusion criteria listed.
		(*b*) *Cohort study*—For matched studies, give matching criteria and number of exposed and unexposed<br>*Case-control study*—For matched studies, give matching criteria and the number of controls per case	N/A	Not applicable: this was an unmatched case-control study.
Variables	7	Clearly define all outcomes, exposures, predictors, potential confounders, and effect modifiers. Give diagnostic criteria, if applicable	3-4	Fracture status, BMI measures, 25OHD, PTH, ALP, calcium, phosphate, calcium intake, season, and mineralization pattern are defined.
Data sources/ measurement	8*	For each variable of interest, give sources of data and details of methods of assessment (measurement). Describe comparability of assessment methods if there is more than one group	3-4	Anthropometry/CDC LMS, blood handling, laboratory measures/reference ranges, and validated calcium questionnaire; common protocol for both groups.
Bias	9	Describe any efforts to address potential sources of bias	3-4, 12	Consecutive screening, comparable-trauma controls, standardized assessment, adjusted models, and bias-related limitations.
Study size	10	Explain how the study size was arrived at	Not reported	No sample-size rationale or calculation is provided.

Continued on next page

Quantitative variables	11	Explain how quantitative variables were handled in the analyses. If applicable, describe which groupings were chosen and why	3-4	BMI z scores/percentiles; prespecified age groups; vitamin D categories; distribution-based tertiles; season categories.
Statistical methods	12	(*a*) Describe all statistical methods, including those used to control for confounding	4	t test/Mann-Whitney U, chi-square, Spearman correlation, and multivariable logistic regression; covariates stated.
		(*b*) Describe any methods used to examine subgroups and interactions	3-4, 9-10	Age-stratified and exploratory 25OHD/calcium-intake tertile analyses.
		(*c*) Explain how missing data were addressed	4	'There were no missing data for the variables included in the reported analyses.'
		(*d*) *Cohort study*—If applicable, explain how loss to follow-up was addressed<br>*Case-control study*—If applicable, explain how matching of cases and controls was addressed<br>*Cross-sectional study*—If applicable, describe analytical methods taking account of sampling strategy	N/A	Not applicable: unmatched case-control design with no follow-up.
		(*e*) Describe any sensitivity analyses	N/A	No sensitivity analyses were reported.
**Results**				
Participants	13*	(a) Report numbers of individuals at each stage of study—eg numbers potentially eligible, examined for eligibility, confirmed eligible, included in the study, completing follow-up, and analysed	4	100 participants were analyzed: 50 fracture cases and 50 controls. Screening-stage counts are not reported.
		(b) Give reasons for non-participation at each stage	Not reported	Reasons for non-participation are not reported.
		(c) Consider use of a flow diagram	Not reported	No participant-flow diagram is included.
Descriptive data	14*	(a) Give characteristics of study participants (eg demographic, clinical, social) and information on exposures and potential confounders	4-5	Table 1 reports demographic, anthropometric, and biochemical characteristics separately for cases and controls.
		(b) Indicate number of participants with missing data for each variable of interest	4	No missing data in the reported analyses.
		(c) *Cohort study*—Summarise follow-up time (eg, average and total amount)	N/A	Not applicable to a case-control study.
Outcome data	15*	*Cohort study*—Report numbers of outcome events or summary measures over time	N/A	Not applicable: cohort-study item.
		Case-control study—Report numbers in each exposure category, or summary measures of exposure	4-7	Table 1 and Figures 1-3 summarize exposures for fracture cases and controls.
		Cross-sectional study—Report numbers of outcome events or summary measures	N/A	Not applicable: cross-sectional-study item.
Main results	16	(*a*) Give unadjusted estimates and, if applicable, confounder-adjusted estimates and their precision (eg, 95% confidence interval). Make clear which confounders were adjusted for and why they were included	4-5, 9-10	Table 1 gives unadjusted comparisons; Tables 3-4 give adjusted coefficients, odds ratios, and 95% CIs. Model covariates are specified.
		(*b*) Report category boundaries when continuous variables were categorized	4, 9-10	Vitamin D category cut-offs are reported; numerical tertile cut-points are not reported.
		(*c*) If relevant, consider translating estimates of relative risk into absolute risk for a meaningful time period	N/A	Not applicable: absolute incidence risk over time cannot be estimated from this case-control design.

Continued on next page

Other analyses	17	Report other analyses done—eg analyses of subgroups and interactions, and sensitivity analyses	7-10	Correlations, seasonal comparison, graphical analyses, and exploratory tertile model.
**Discussion**				
Key results	18	Summarise key results with reference to study objectives	10-12	Discussion and Conclusions summarize adiposity, 25OHD, and mineralization findings.
Limitations	19	Discuss limitations of the study, taking into account sources of potential bias or imprecision. Discuss both direction and magnitude of any potential bias	12	Limitations: sample size/single-region design, post-injury ALP, residual confounding, absent BMD, and calcium-intake measurement bias.
Interpretation	20	Give a cautious overall interpretation of results considering objectives, limitations, multiplicity of analyses, results from similar studies, and other relevant evidence	10-12	Results are interpreted in relation to the objectives, limitations, and existing pediatric evidence, with cautious clinical implications.
Generalisability	21	Discuss the generalisability (external validity) of the study results	12	Single-region design limits generalizability; larger longitudinal validation is recommended.
**Other information**				
Funding	22	Give the source of funding and the role of the funders for the present study and, if applicable, for the original study on which the present article is based	12	'This research received no external funding.'

*Give information separately for cases and controls in case-control studies and, if applicable, for exposed and unexposed groups in cohort and cross-sectional studies.

Note: An Explanation and Elaboration article discusses each checklist item and gives methodological background and published examples of transparent reporting. The STROBE checklist is best used in conjunction with this article (freely available on the Web sites of PLoS Medicine at http://www.plosmedicine.org/, Annals of Internal Medicine at http://www.annals.org/, and Epidemiology at http://www.epidem.com/). Information on the STROBE Initiative is available at www.strobe-statement.org.
