# Supplementary material for: BMI, Vitamin D Status, and Biochemical Markers of Mineralization in Children with Low-Energy Fractures: A Prospective Case–Control Study
Source: Children (Basel). 2026 Sep 20;13(9):1276. doi: 10.3390/children13091276 (PMC13605413; doi:10.3390/children13091276)
Supplement: Supplementary file 1 [file children-13-01276-s001.zip › Supplementary Table S1.pdf]

Supplementary Table S1. Exploratory multivariable binomial logistic regression of fracture status using cohort-derived tertiles of serum 25OHD and calcium intake.

Predictor	β	SE	Z	p	Adjusted OR	95% CI lower	95% CI upper
**Intercept**	−2.913	1.270	−2.294	0.022	0.054	0.005	0.654
**25OHD tertile**							
**Medium vs. high**	0.303	0.558	0.543	0.587	1.354	0.453	4.043
**Low vs. high**	1.098	0.554	1.982	0.048	2.998	1.012	8.878
**Calcium-intake tertile**							
**Medium vs. low**	−0.859	0.573	−1.499	0.134	0.423	0.138	1.303
**High vs. low**	−0.749	0.530	−1.414	0.157	0.473	0.167	1.336
**BMI, kg/m²**	0.199	0.076	2.611	0.009	1.220	1.051	1.417
**Age, years**	−0.066	0.076	−0.876	0.381	0.936	0.807	1.086

Note: The dependent variable was fracture status, coded as case = 1 and control = 0; therefore, an OR greater than 1 indicates higher odds of fracture. High 25OHD and low calcium intake were the reference categories. The tertile boundaries were derived from the study sample; consequently, this analysis should be considered exploratory. OR, odds ratio; CI, confidence interval; 25OHD, 25-hydroxyvitamin D.
